# Janus Kinase 3 (JAK3): A Critical Conserved Node in Immunity Disrupted in Immune Cell Cancer and Immunodeficiency

**DOI:** 10.3390/ijms25052977

**Published:** 2024-03-04

**Authors:** Clifford Liongue, Tarindhi Ratnayake, Faiza Basheer, Alister C. Ward

**Affiliations:** 1 School of Medicine, Deakin University, Geelong, VIC 3216, Australia; c.liongue@deakin.edu.au (C.L.); s222416671@deakin.edu.au (T.R.); faiza.basheer@deakin.edu.au (F.B.); 2 The Institute for Mental and Physical Health and Clinical Translation (IMPACT), Deakin University, Geelong, VIC 3216, Australia

**Keywords:** cytokine, cytokine receptor, immunity, immunodeficiency, JAK3, leukemia

## Abstract

The Janus kinase (JAK) family is a small group of protein tyrosine kinases that represent a central component of intracellular signaling downstream from a myriad of cytokine receptors. The JAK3 family member performs a particularly important role in facilitating signal transduction for a key set of cytokine receptors that are essential for immune cell development and function. Mutations that impact JAK3 activity have been identified in a number of human diseases, including somatic gain-of-function (GOF) mutations associated with immune cell malignancies and germline loss-of-function (LOF) mutations associated with immunodeficiency. The structure, function and impacts of both GOF and LOF mutations of JAK3 are highly conserved, making animal models highly informative. This review details the biology of JAK3 and the impact of its perturbation in immune cell-related diseases, including relevant animal studies.

## 1. Introduction

Cytokine receptor signaling represents a pivotal mode of cell-to-cell regulation that substantially impacts immune cell development and function [1]. Expressed on the surface of specific immune cell subsets, the activation of specific cytokine receptors by their respective cytokine can mediate an array of cellular responses, which can include lineage commitment, differentiation, proliferation, survival or functional activation [2]. Critical for transducing cytokine binding into the appropriate intracellular signals are protein tyrosine kinases (PTKs) called Janus kinases (JAKs) that are bound to the cytoplasmic domain of relevant cytokine receptors. Amongst these, the JAK3 protein is exclusive to a specific family of cytokine receptors that play multiple pivotal roles in immunity. Not surprisingly, therefore, mutations in JAK3 have been associated with a number of important immune cell diseases, with somatic gain-of-function (GOF) mutations identified in various immune cell malignancies and germline loss-of-function (LOF) mutations causative for severe combined immunodeficiency (SCID) [3]. This review describes the underlying biology of JAK proteins, and specifically the role of JAK3 in immune cell development and function. It then details how the JAK3 protein is disrupted by various mutations to mediate relevant immune cell diseases, including its cross-species conservation.

## 2. JAK Biology

### 2.1. Structure

**Figure 1:**
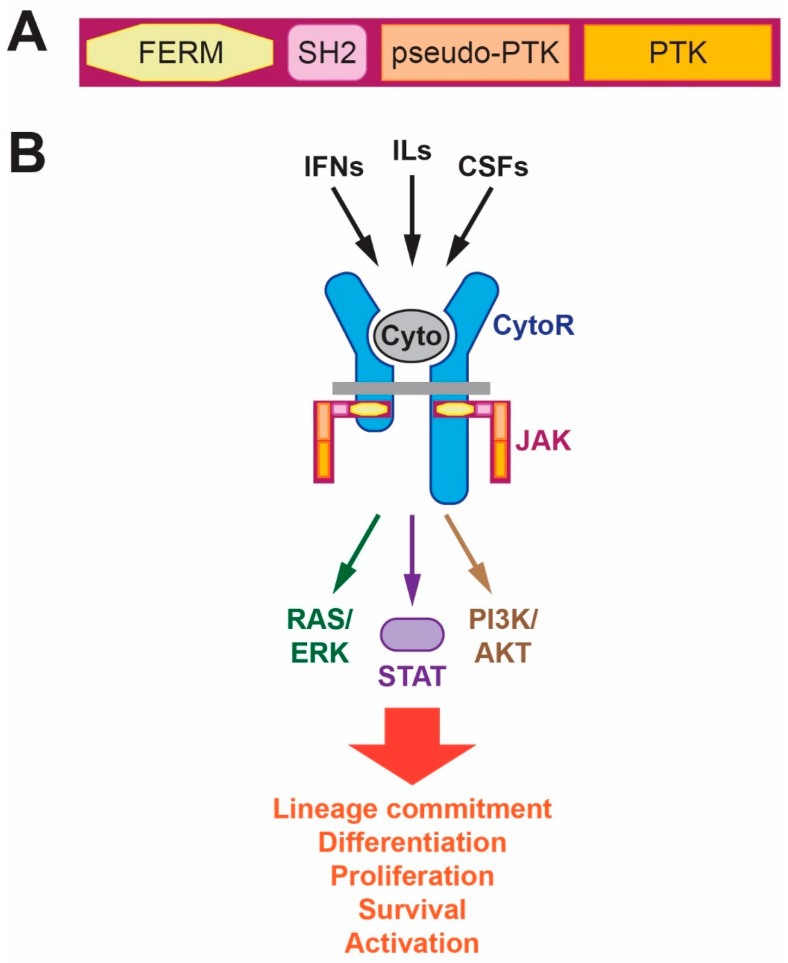
Structure and function of the JAK proteins. (**A**) Diagram of a JAK protein showing the four domains common in all family members: FERM (yellow), SH2 (pink), pseudo-PTK (fawn) and PTK (orange). (**B**) Schematic representation of major cytokine receptor signaling pathways, highlighting the key role for JAK proteins. Various cytokines (Cyto) bind to their specific cytokine receptor complex (CytoR) to activate associated JAK proteins that mediate extensive tyrosine phosphorylation that initiates multiple intracellular signaling pathways to facilitate a variety of cellular impacts. Abbreviations: AKT: Ak strain transforming; CSF: colony-stimulating factor; ERK: extracellular-regulated kinase; FERM: four-point-one, ezrin, radixin, moesin; IFN: interferon; IL: interleukin; PI3K: phosphatidyl inositol 3′-kinase; PTK: protein tyrosine kinase; RAS: rat sarcoma; SH2: SRC homology 2; STAT: signal transducer and activator of transcription.

The JAK protein family consists of four members in mammals: JAK1, JAK2, JAK3 and the alternatively named tyrosine kinase 2 (TYK2) [4]. Each of the JAK family members consists of the same unique structural architecture comprising four domains. At the N-terminus is a so-called four-point-one, ezrin, radixin, moesin (FERM) domain that lies in juxtaposition with a modified SRC homology 2 (SH2) domain that collectively facilitate binding of the JAK protein to the cytoplasmic domain of relevant cytokine receptors. Adjacent to this lies a central pseudo-protein tyrosine kinase (pseudo-PTK) domain, which shows considerable homology to PTK domains but possesses no enzymatic activity and instead exerts a regulatory function on the protein. At the C-terminus, in contrast, is a classical PTK domain that provides the critical catalytic function for each JAK protein [5] (Figure 1A). This identical domain structure, with a high level of conservation at the amino acid level, is found in JAK proteins across a myriad of higher vertebrates [6].

### 2.2. Function

Cytokine receptor signaling plays a number of pivotal roles in development and homeostasis but with particularly important functions in the generation and function of blood and immune cells. The majority of these signal through JAK kinases associated with the membrane-proximal region of their cytoplasmic domains, which activate a variety of intracellular signaling pathways. These notably include the latent signal transducer and activator of transcription (STAT transcription factors but also include those involving the rat sarcoma/extracellular-regulated kinase (RAS/ERK) and phosphatidyl inositol 3-kinase/Ak strain transforming (PI3K/AKT) pathways [7,8,9]. Collectively, these pathways elicit the full gamut of cellular responses following cytokine receptor activation from lineage commitment and differentiation to cell proliferation and survival to functional activation (Figure 1B).

## 3. JAK3 and Its Role in Immune Development

### 3.1. Gene Expression

The human *JAK3* gene, located at 19p13.11, is unique amongst the four *JAK* genes in having restricted expression in hematopoietic cells, particularly within those belonging to the lymphoid compartment, although this also extends to certain myeloid cell populations [10,11]. The mouse *Jak3* gene similarly displays its highest expression in the thymus and spleen, which is elevated in double-negative thymocytes [12]. Zebrafish *jak3* has also been shown to be expressed in the embryonic thymus as well as adult hematopoietic and lymphoid tissues [13].

### 3.2. Role in Cytokine Receptor Signaling

**Figure 2:**
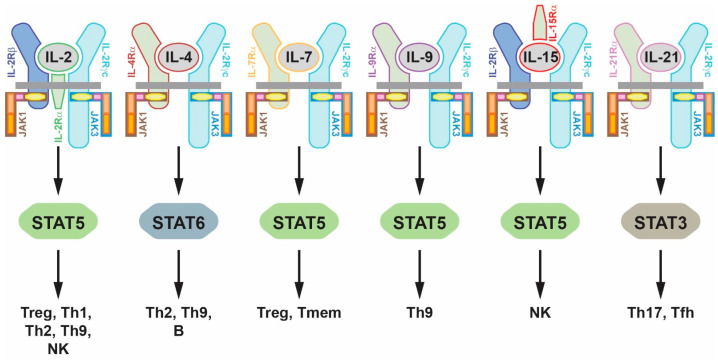
Central role for JAK3 in signaling via the interleukin 2 cytokine receptor family. Schematic representation of the cytokine receptor signaling complexes for the interleukin 2 (IL-2) family of cytokines (gray ellipses), including IL-2 (green edge), IL-4 (brown edge), IL-7 (orange edge), IL-9 (purple edge), IL-15 (red edge) and IL-21 (pink edge), with their respective ligand-specific chains (green, matching edges), the shared IL-2 receptor gamma common (IL-2Rγc) signaling chain (light blue), with an additional shared IL-2 receptor beta (IL-2Rβ) chain (dark blue) in two cases. Associated with the IL-2Rγc chain is JAK3 (blue edge) and associated with one of the alternative chains is JAK1 (brown edge). These cytokine receptor complexes activate multiple signaling pathways but particularly the indicated STAT proteins, STAT3 (brown), STAT5 (green) and STAT6 (blue). Below this is indicated the major cell lineage impacted by each cytokine receptor signaling complex. Abbreviations: IL: interleukin; NK: natural killer; Tfh: follicular helper T cell; Th: helper T cell; Tmem: memory T cell; Treg: regulatory T cell.

The development and function of the various lymphoid lineages is largely driven by cytokines that act via the interleukin 2 receptor (IL-2R) cytokine receptor family, of which IL-2R, IL-4R type I, IL-7R, IL-9R, IL-15R and IL-21R are members [14]. Each of these cytokine receptor complexes possesses a unique ligand-specific chain, but shares a common signal transducing chain, called IL-2R gamma common (IL-2Rγc), and in some cases a third IL-2R beta (IL-2Rβ) chain [15,16]. JAK3 exclusively associates with the IL-2Rγc chain, whereas JAK1 associates with an alternate chain [17] (Figure 1B). JAK1 has been demonstrated to exert a dominant function over JAK3 with respect to signaling from these cytokine receptor complexes [18,19] with JAK3 playing a secondary but still important role. Collectively, these two JAKs activate multiple intracellular signaling pathways to mediate the appropriate cellular responses for each cytokine receptor. These include signal transducer and activator of transcription (STAT) proteins, principally STAT5 downstream of IL-2R, IL-7R, IL-9R and IL-15R, STAT6 downstream of IL-4R type I and STAT3 downstream of IL-21R, which in concert with other pathways [7,8,9] impacts specific cell lineages (Figure 2).

IL-2R stimulates T cell proliferation and is critical for regulatory T cell (Treg) development, promoting differentiation of the helper T cell (Th) populations Th1, Th2 and Th9 but antagonizing differentiation of Th17 and follicular helper T cell (Tfh) populations as well as mediating natural killer (NK) cell proliferation and enhancing B cell function. IL-4R type I also promotes Th2 and Th9 differentiation, but it additionally plays a major role in B cell differentiation, including immunoglobulin (Ig) switching, and macrophage differentiation. IL-7R stimulates the generation of Treg and memory T cell (Tmem) populations and contributes to overall T cell homeostasis. IL-9R is a major promoter of Th9 differentiation and also facilitates mast cell proliferation and mucus production. IL-15R is the principal mediator of NK cell development, proliferation and survival but also contributes to Tmem differentiation while antagonizing Th17 differentiation. Finally, IL-21R promotes Th17 and Tfh development and enhances the antitumor actions of lymphoid cells but antagonizes Th9 and B cell differentiation and Ig production [14,20].

## 4. JAK3 GOF Mutations in Immune Cell Cancers

### 4.1. Human Disease Specificity

Somatic JAK3 GOF mutations have been identified in the context of a diverse spread of human immune cell cancers. These mutations are most significant across a range of T cell malignancies, notably including T cell acute lymphoblastic leukemia (T-ALL) [21,22,23], cutaneous T cell lymphoma (CTCL) [7,24], early T cell precursor acute lymphoblastic leukemia (ETP-ALL) [25], NK/T cell lymphoma (NKTCL) [26], T cell prolymphocytic leukemia (T-PLL) [27,28] and enteropathy-associated T cell lymphoma (EATL) [29]. Germline JAK3 GOF mutations have also been identified in the context of familial chronic lymphoproliferative disorder of NK cells (CLPD-NKs) [30]. JAK3 has further been observed to be constitutively activated in a range of lymphoid malignancies even when not mutated, such as a cohort of peripheral T cell lymphoma (PTCL) patients positive for ALK [31], underpinning its central role in T cell malignancies.

Somatic JAK3 GOF mutations have also been identified in cancers affecting other hematopoietic cell lineages, such as acute megakaryoblastic leukemia (AMKL) [32,33,34], juvenile myelomonocytic leukemia (JMML) [35] and B cell precursor acute lymphoblastic leukemia (BCP-ALL) [36]. A large number of JAK3 mutations have additionally been found in a variety of solid tumors, most notably including lung cancer [37] and high-grade serous ovarian cancer [38]. Analysis of TCGA Research Network data additionally revealed the mutations in solid tumors to be a mix of GOF, LOF and likely benign. *JAK3* copy number changes were also evident with copy number gain most prevalent in adenoma/adenocarcinoma, ovarian cancer and glioblastoma, while copy number loss was highest in cancers of the bronchus and lungs.

### 4.2. Mode of Action

**Figure 3:**
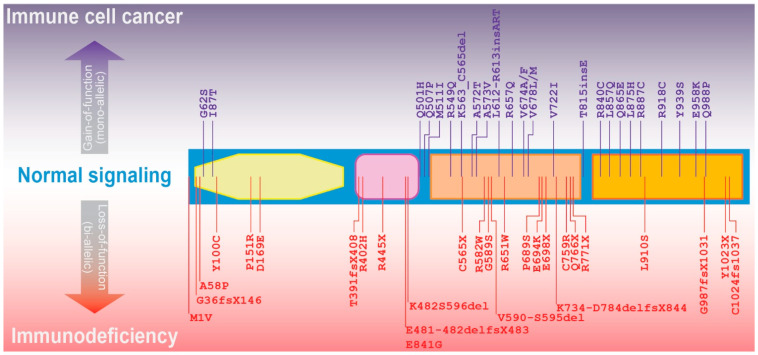
JAK3 mutations associated with immune cell diseases. Schematic of the JAK3 protein and its constituent FERM (yellow), SH2 (pink), pseudo-PTK (fawn), and PTK (orange) domains, showing representative gain-of-function mutations (purple, above), typically mono-allelic and somatic, associated with immune cell cancers, and loss-of-function (red, below) mutations, typically bi-allelic and germline, associated with immunodeficiency [45,46,47,48,49,50,51,52,53,54,55,56,57,58].

A large number of mutations in JAK3 have been identified in immune cell cancers, the majority being within the pseudo-PTK and PTK domains, with the most common being M511I, A573V and R657A in the pseudo-PTK domain, although no clear correlation of specific mutations with cancer type has been identified [39] (Figure 3). A JAK3–INSL3 fusion transcript has also been reported in CTCL [40]. JAK3 GOF mutations have been collectively found to result in constitutive tyrosine-phosphorylation and the ability to elicit factor-independent growth of relevant cell lines [26,32,41]. As such, these mutations are typically thought of as bypassing normal control mechanisms leading to the chronic activation of pathways normally activated downstream of those cytokine receptor complexes with which JAK3 typically interacts [41,42]. However, so-called ‘non-canonical’ mechanisms have also been identified with JAK3 GOF proteins found in the nucleus of CTCL cells where they could phosphorylate histone H3 and interact with RNA polymerase III [43].

### 4.3. Animal Models

The transplantation of mouse bone marrow cells overexpressing human JAK3 A572V into lethally irradiated mice produced a lymphoproliferative disorder with megakaryocytic hyperplasia [7]. Both this and another pseudo-PTK domain mutant, JAK3 M511I, were also shown to induce a transplantable T-ALL-like disease with long latency, which was characterized by the ligand-independent proliferation of T cells, while the PTK domain mutant L857Q displayed splenomegaly and lymphadenopathy without the peripheral increase in T cells [41]. A mouse knock-in model that expressed an A572V mutant of mouse Jak3 from its native promoter exhibited a much milder disease with CD8+ T cells expanding over time and evidence of minor skin pathology [17]. A zebrafish Jak3 A573V knock-in mutant also showed enhanced lymphopoiesis over the lifespan, with a sustained elevation of T and NK cells, but not B cells [44]. No evidence of malignancy was identified in either study [17,44], consistent with JAK3 GOF mutations being able to cause a mild lymphoproliferative disorder rather than overt leukemia, which requires additional genetic events.

### 4.4. Interactions with Other Genes and Mutations

JAK3 GOF mutations typically do not act autonomously, with other signaling components critically needed to facilitate their cellular effects. Amongst these, the IL-2Rγc chain has been shown to be required for both downstream signaling and factor-independent growth mediated by JAK3 GOF mutants [59], with both IL-2Rγc and JAK1 needed for the maximal activation of downstream signaling proteins, including STAT5 phosphorylation [19,41,42]. The kinase activity of JAK1 has been demonstrated to be essential to facilitate this [18,19,42] with JAK1 proteins being constitutively activated in the presence of JAK3 mutants [42,47]. STAT5 proteins have also been shown to be constitutively activated by JAK3 GOF mutants [42,47]. Genetic analyses in animal models have confirmed the importance of these other pathway components. Thus, both IL-2Rγc and JAK1 were found to be essential for zebrafish Jak3 A573V to mediate embryonic T cell expansion [44] with a similar result observed for IL-2Rγc in the context of the mouse Jak3 A572V knock-in model [17]. Zebrafish Jak3 A573V also caused an overactivation of the Stat5.1 protein, which contributed to the increase in embryonic T cells [44]. It has been suggested that the presence of wild-type JAK3 suppresses the effects of JAK3 GOF mutants, explaining why patients are often homozygous or compound heterozygous for JAK3 mutations [47,60]. However, zebrafish carrying two copies of the Jak3 A573V allele displayed similar impacts on lymphopoiesis as those with a single copy, although homozygote mutants did exhibit reduced survival [44]. Importantly, some JAK3 mutants, particularly those involving the PTK domain such as L857P and Q988P, did not show dependence on JAK1 [41,49].

In terms of their role in the etiology of human immune cell cancers, evidence suggests that JAK3 GOF mutations act as ‘disease driver’ mutations within the context of T cell malignancies, with ‘co-operating’ mutations essential for JAK3-mediated leukemic transformation [22,27,41]. A number of classes of co-operating mutations have been identified, which include transcription factors, epigenetic regulators and other signaling components as well as chromosomal aberrations that likely impact multiple genes (Table 1).

Several hematopoietic transcription factors have been found to co-operate with JAK3 GOF mutations across a range of leukemia types. These commonly include RUNX1 LOF mutations [61] and HOXA gene overexpression [62]. Thus, around one-quarter of T-ALL patients harboring JAK3 GOF mutations also possessed RUNX1 LOF mutations, with co-mutation enriched in the ETP-ALL cohort, while JAK3 GOF and RUNX LOF mutations synergized in a murine T-ALL model [61]. In contrast, the overexpression of *HOXA* genes, particularly *HOXA9*, has been observed concurrently with JAK3 GOF mutations in T-ALL with *HOXA9* overexpression co-operating with the JAK3 M511I mutation to elicit a short latency, aggressive leukemia in mice [66]. This correlated with enhanced STAT5 transcriptional activity and the co-occupation of similar genomic loci with HOXA9 [66]. JAK3 GOF mutations are also associated with LOF mutations of epigenetic regulators, such as PHF6 [63] and SUZ12 [64]. In T-ALL, over 40% of patients with JAK3 GOF mutations also possessed PHF6 LOF mutations, with this cohort showing reduced survival, while PHF6 inactivation in mice accelerated cell transformation mediated by JAK3 M511I, inducing an aggressive form of leukemia [63]. SUZ12 LOF mutations have also been associated with JAK3 in T-ALL with an ablation of SUZ12 co-operating with JAK3 M511I to drive transformation in a mouse pro-T cell ex vivo model [64]. Mutations in other signaling components additionally co-operate with JAK3 GOF mutations. Principal amongst these are concurrent JAK1 GOF mutations observed in several T cell malignancies [22,23,26,27,29,39]. JAK1 GOF and JAK3 GOF mutations have been shown to synergize in vitro [18] with concurrent mutations increasing downstream STAT5 activation [65]. STAT5B GOF mutations have also been identified concurrently with JAK3 GOF mutations in ALL [23,60] and EATL [29]. Concurrent GOF mutations in other signaling proteins, such as IL-7RA [22] and RAS pathway components [29], may also be important. However, such associations exhibit cell lineage specificity, with mutations in JAK3, JAK1 and STAT5B reported to be mutually exclusive in T-PLL, for example [28,48]. Finally, JAK3 GOF mutations were initially identified in the context of Down syndrome-associated AMKL [32,33]. Subsequent experiments in a mouse Jak3 A572V knock-in line have demonstrated that partial trisomy 21 enhanced the aggressiveness of cutaneous T-cell lymphoma (CTCL)-like phenotypes, making it a fully penetrant, lethal disease [17].

In the context of other malignancies, JAK3 GOF mutations are believed to represent co-operating mutations. Thus, in B cell malignancies, they act in concert with alternative disease driver mutations, including PAX5 fusions [67], SPI1 deletions [68] and IRF4 deficiency [36]. In JMML, they also act in concert with alternative disease driver mutations to contribute to disease progression, being associated with poor clinical outcomes [35].

### 4.5. Therapeutic Approaches

A variety of small molecule inhibitors targeting JAK proteins have been developed that are applicable to JAK3 GOF-mediated disease [69,70], although the viability of JAK3-specific inhibitors has been questioned due to the dominant role played by JAK1 in signal transduction [18], and so attention has focused on pan-JAK inhibitors. Ruxolitinib, which targets only JAK1 and JAK2 and is approved for use in myeloproliferative neoplasms [71], has displayed effectiveness against JAK3 pseudo-PTK domain GOF mutations [72]. However, it has proven less effective in PTK domain mutants, such as Q988P, which are more sensitive to JAK3-specific inhibitors [42,49]. Moreover, the use of Ruxolitinib to treat JAK1 GOF mutations has been shown to expand clones containing JAK3 mutations [39]. Tofacitib, which targets JAK3 as well as both JAK1 and JAK2, and is approved for use in rheumatoid and psoriatic arthritis and ulcerative colitis [71], has been shown to be highly effective in cell line models of various JAK3 GOF mutations [26,42,73,74]. Notably, phosphoproteome analysis revealed that STAT5 phosphorylation was the most impacted by both Tofacitib and Ruxolitinib, but this was highest for Tofacitib [74]. Tofacitib was also impactful in lymphocyte expansion in both mouse Jak3 A572V [17] and zebrafish Jak3 A573V [44] models. Furthermore, efficacy for this drug has been demonstrated in relevant human clinical trials [75]. A JAK3 inhibitor, PRN371, was shown to be effective in NKTL cell lines and mouse models [76]. The JAK3/TEC-selective inhibitor PF-06651600, being trialed for several inflammatory bowel and dermatological diseases [71], also has potential. JAK3 inhibitors have also proven effective in cases of PTCL in which JAK3 is constitutively activated but not mutated [31]. Of note, co-operating JAK1 and JAK3 mutations are associated with enhanced resistance to JAK inhibitors generally [65], suggesting the need for alternative strategies. Of relevance, potential synergy has been noted between JAK inhibitors and inhibitors of other pathways, including MEK and BCL2 [47].

## 5. JAK3 LOF Mutations in Immunodeficiency

### 5.1. Human Disease Specificity

JAK3 LOF mutations cause an autosomal-recessive form of severe combined immune deficiency (SCID), comprising approximately 5% of total SCID [77]. SCID is associated with defects in T cell differentiation leading to low numbers of mature T cells, being defined as <0.05 × 10^9^ autologous T cells/L [78]. This results in disease symptoms characterized by recurrent respiratory tract infections, pneumonia meningitis and failure to thrive with patients typically presenting with hypogammaglobulinemia [79]. There is an extremely high risk of severe, disseminated infections following live-attenuated vaccine inoculation that often proves fatal, particular the Bacille Camette–Guerin (BCG) vaccine based on *Mycobacterium bovis* [80].

### 5.2. Mode of Action

Close to 100 different SCID-causing JAK3 mutations have been identified, most of which directly impact the coding region [77,78], although rare mutations impacting splicing have also been described [81]. These can affect all domains of the JAK3 protein but particularly in the pseudo-PTK domain, including a large number that lead to truncations of the encoded protein (Figure 3). These JAK3 LOF mutations impair signaling from the suite of IL-2Rγc-utilizing receptors, which results in the severe impacts across all lymphoid lineages [51,82]. Moreover, patients harboring these mutations present with lymphoid tissue hypoplasia as a consequence of severely reduced T and NK cells, and while B cells are generated, their functionality is compromised [83,84]. Patients with hypomorphic mutations, such as the missense E481G mutation with the SH2 domain, typically harbor a milder SCID phenotype [85]. Somatic JAK3 mutations have also been identified in cases of NK cell enteropathy [57].

### 5.3. Animal Models

Jak3 LOF mice also possessed severely reduced numbers of B and T cells [86] as well as specific innate lymphoid populations including NK cells [87]. They further exhibited impaired myelopoiesis that impacts the differentiation of both monocytes and neutrophils [88]. Zebrafish Jak3 LOF mutants displayed a severe reduction in T cells during embryonic hematopoiesis [13,89], while in adults both T and NK cells were decreased [13,90], with evidence of disrupted B cell development as well [13]. These Jak3 LOF mutants additionally developed an invasive lymphoid leukemia during adulthood suggesting that tumor immunity was abrogated, consistent with the reduced NK cells observed, and also showed perturbed neutrophil homeostasis with circulating numbers increased at the expense of those in the kidney [13].

### 5.4. Treatment of JAK3-Mediated SCID

Hematopoietic stem cell (HSC) transplantation is curative for SCID patients with JAK3 LOF and other related mutations [91], but while it represents an effective means to reconstitute T cell immunity but is less effective for B cells and NK cells [79]. Transplant recipients display defects in mucosal immunity with nasophyrangeal dysbiosis over the long term, although immunoglobulin replacement therapy can aid with these presentations [92]. Pre-transplant conditioning can improve outcomes, with patients exhibiting increased CD4+ T cells and B cells, obviating the need for IgG replacement therapy [93]. Gene therapy of HSC using induced pluripotent stem cell (iPSC) technology is an attractive alternative option, but a number of significant hurdles remain [94].

## 6. Wider Pathway Conservation

The conservation observed with respect to the JAK3 structure, function and role in pathogenesis also extends to other relevant cytokine receptor signaling components [2,6]. For example, patients with LOF mutations in the human IL2Rγc-chain presented with X-linked SCID (X-SCID) [95]. Il-2rγc knockout mice also developed SCID, albeit more severe with B cell numbers additionally decreased [86,96], whereas an ablation of Il-2rγc in zebrafish resulted in T−B+NK−SCID similar to humans [97]. Furthermore, patients harboring LOF mutations in the IL-7 receptor alpha (IL-7Rα) chain display a T-B+NK+ SCID phenotype [98,99]. Mice with IL-7Rα deficiency again possessed a more severe SCID that included B cell involvement [100,101]. Zebrafish carrying an inactivating mutation of Il-7rα exhibited reduced T cells, but unaffected B cells, again similar to humans, with NK cells not characterized in this study [89]. STAT5B-deficient humans also displayed immunodeficiency and growth defects [102], which closely resembled those observed in Zebrafish Stat5.1 LOF mutants [103]. In contrast, an activating zebrafish Stat5.1 N649H mutant impacted both lymphoid and myeloid lineages [104], replicating features of the STAT5B N649H-mediated disease observed in mice [105]. This high conservation makes animal models particularly suited to explore JAK3 biology, pathology and potential therapeutic strategies.

## 7. Conclusions

JAK proteins were named after the two-faced Roman god Janus based on structural considerations, since they possessed pseudo-kinase and kinase domains in juxtaposition, However, this eponym has also proven apt with respect to both its normal function and role in disease. For Janus is additionally the god of gates, with JAK3 representing the gate-keeper controlling the transition between inactive and active states for cytokine receptor signaling, but also the god of beginnings and ends, with JAK3 LOF mutations preventing cytokine receptor signaling from commencing, while JAK3 GOF mutations interfere with its termination. The conservation of this gatekeeper and those signaling components with which it interacts has allowed animal models to act as key platforms to understand JAK3 biology and pathology with considerable future promise for the development of treatment strategies for relevant diseases. For example, there are clear opportunities to use the zebrafish Jak3 LOF model to further understand the role of JAK3 in neutrophil homeostasis and susceptibility to lymphoid malignancy with the JAK3 GOF model amenable to investigating co-operation with other genes as well as pharmacological testing. Conversely, the zebrafish JAK3 LOF model can be applied as a xenotransplantation platform to examine JAK3 GOF-mediated cancers in a patient-specific manner, underpinning the development of patient-centered therapeutic approaches.

## Figures and Tables

**Table 1:** Examples of JAK3 co-operating gene mutations.

Functional Class	Protein	Mutation Type	Disease	References
Transcription factor	RUNX1	LOF	ETP-ALL	[61]
	HOXA9	GOF	T-ALL	[62]
Epigenetic modifier	PHF6	LOF	T-ALL	[63]
	SUZ12	LOF	T-ALL	[64]
Signaling component	JAK1	GOF	T-PLL	[27,65]
	STAT5B	GOF	T-ALL, EATL	[29,60]
Multiple	Various on chromosome 21	↑ copy no. (trisomy)	CTCL	[17]

Abbreviations: CTCL: cutaneous T cell lymphoma; EATL: enteropathy-associated T cell lymphoma; ETP-ALL: early T cell precursor acute lymphoblastic leukemia; GOF: gain of function; LOF: loss of function; T-ALL: T cell acute lymphoblastic leukemia; T-PLL: T cell prolymphocytic leukemia; ↑: increased.

## References

[1] O’Shea J.J., Holland S.M., Staudt L.M. (2013). JAKs and STATs in immunity, immunodeficiency, and cancer. N. Engl. J. Med..

[2] Liongue C., O’Sullivan L.A., Trengove M.C., Ward A.C. (2012). Evolution of JAK-STAT pathway components: Mechanisms and role in immune system development. PLoS ONE.

[3] Yamaoka K., Saharinen P., Pesu M., Holt V.E., Silvennoinen O., O’Shea J.J. (2004). The Janus kinases (Jaks). Genome Biol..

[4] Philips R.L., Wang Y., Cheon H., Kanno Y., Gadina M., Sartorelli V., Horvath C.M., Darnell J.E., Stark G.R., O’Shea J.J. (2022). The JAK-STAT pathway at 30: Much learned, much more to do. Cell.

[5] Stark G.R., Darnell J.E. (2012). The JAK-STAT pathway at twenty. Immunity.

[6] Liongue C., Ward A.C. (2007). Evolution of class I cytokine receptors. BMC Evol. Biol..

[7] Cornejo M.G., Kharas M.G., Werneck M.B., Le Bras S., Moore S.A., Ball B., Beylot-Barry M., Rodig S.J., Aster J.C., Lee B.H. (2009). Constitutive JAK3 activation induces lymphoproliferative syndromes in murine bone marrow transplantation models. Blood.

[8] Malek T.R., Castro I. (2010). Interleukin-2 receptor signaling: At the interface between tolerance and immunity. Immunity.

[9] Liao W., Lin J.X., Leonard W.J. (2013). Interleukin-2 at the crossroads of effector responses, tolerance, and immunotherapy. Immunity.

[10] Kawamura M., McVicar D.W., Johnston J.A., Blake T.B., Chen Y.Q., Lal B.K., Lloyd A.R., Kelvin D.J., Staples J.E., Ortaldo J.R. (1994). Molecular cloning of L-JAK, a Janus family protein-tyrosine kinase expressed in natural killer cells and activated leukocytes. Proc. Natl. Acad. Sci. USA.

[11] Witthuhn B.A., Silvennoinen O., Miura O., Lai K.S., Cwik C., Liu E.T., Ihle J.N. (1994). Involvement of the Jak-3 Janus kinase in signalling by interleukins 2 and 4 in lymphoid and myeloid cells. Nature.

[12] Gurniak C.B., Berg L.J. (1996). Murine JAK3 is preferentially expressed in hematopoietic tissues and lymphocyte precursor cells. Blood.

[13] Basheer F., Lee E., Liongue C., Ward A.C. (2022). Zebrafish model of severe combined immunodeficiency (SCID) due to JAK3 mutation. Biomolecules.

[14] Leonard W.J., Lin J.X., O’Shea J.J. (2019). The gammac family of cytokines: Basic biology to therapeutic ramifications. Immunity.

[15] Leonard W.J., Lin J.-X. (2000). Cytokine receptor signaling pathways. J. Allergy Clin. Immunol..

[16] Lin J.X., Leonard W.J. (2000). The role of Stat5a and Stat5b in signaling by IL-2 family cytokines. Oncogene.

[17] Rivera-Munoz P., Laurent A.P., Siret A., Lopez C.K., Ignacimouttou C., Cornejo M.G., Bawa O., Rameau P., Bernard O.A., Dessen P. (2018). Partial trisomy 21 contributes to T-cell malignancies induced by JAK3-activating mutations in murine models. Blood Adv..

[18] Haan C., Rolvering C., Raulf F., Kapp M., Druckes P., Thoma G., Behrmann I., Zerwes H.G. (2011). Jak1 has a dominant role over Jak3 in signal transduction through gammac-containing cytokine receptors. Chem. Biol..

[19] Raivola J., Hammaren H.M., Virtanen A.T., Bulleeraz V., Ward A.C., Silvennoinen O. (2018). Hyperactivation of oncogenic JAK3 mutants depend on ATP binding to the pseudokinase domain. Front. Oncol..

[20] Lin J.X., Leonard W.J. (2018). The common cytokine receptor gamma chain family of cytokines. Cold Spring Harb. Perspect. Biol..

[21] Mullighan C.G., Zhang J., Harvey R.C., Collins-Underwood J.R., Schulman B.A., Phillips L.A., Tasian S.K., Loh M.L., Su X., Liu W. (2009). JAK mutations in high-risk childhood acute lymphoblastic leukemia. Proc. Natl. Acad. Sci. USA.

[22] Bains T., Heinrich M., Loriaux M., Beadling C., Nelson D., Warrick A., Neff T., Tyner J., Dunlap J., Corless C. (2012). Newly described activating JAK3 mutations in T-cell acute lymphoblastic leukemia. Leukemia.

[23] Atak Z.K., Gianfelici V., Hulselmans G., De Keersmaecker K., Devasia A.G., Geerdens E., Mentens N., Chiaretti S., Durinck K., Uyttebroeck A. (2013). Comprehensive analysis of transcriptome variation uncovers known and novel driver events in T-cell acute lymphoblastic leukemia. PLoS Genet..

[24] McGirt L.Y., Jia P., Baerenwald D.A., Duszynski R.J., Dahlman K.B., Zic J.A., Zwerner J.P., Hucks D., Dave U., Zhao Z. (2015). Whole-genome sequencing reveals oncogenic mutations in mycosis fungoides. Blood.

[25] Zhang J., Ding L., Holmfeldt L., Wu G., Heatley S.L., Payne-Turner D., Easton J., Chen X., Wang J., Rusch M. (2012). The genetic basis of early T-cell precursor acute lymphoblastic leukaemia. Nature.

[26] Koo G.C., Tan S.Y., Tang T., Poon S.L., Allen G.E., Tan L., Chong S.C., Ong W.S., Tay K., Tao M. (2012). Janus kinase 3–activating mutations identified in natural killer/T-cell lymphoma. Cancer Disc..

[27] Bellanger D., Jacquemin V., Chopin M., Pierron G., Bernard O., Ghysdael J., Stern M. (2014). Recurrent JAK1 and JAK3 somatic mutations in T-cell prolymphocytic leukemia. Leukemia.

[28] Lopez C., Bergmann A.K., Paul U., Murga Penas E.M., Nagel I., Betts M.J., Johansson P., Ritgen M., Baumann T., Aymerich M. (2016). Genes encoding members of the JAK-STAT pathway or epigenetic regulators are recurrently mutated in T-cell prolymphocytic leukaemia. Br. J. Haematol..

[29] Nicolae A., Xi L., Pham T.H., Pham T.A., Navarro W., Meeker H.G., Pittaluga S., Jaffe E.S., Raffeld M. (2016). Mutations in the JAK/STAT and RAS signaling pathways are common in intestinal T-cell lymphomas. Leukemia.

[30] Lesmana H., Popescu M., Lewis S., Sahoo S.S., Goodings-Harris C., Onciu M., Choi J.K., Takemoto C., Nichols K.E., Wlodarski M. (2020). Germline gain-of-function mutation in familial chronic lymphoproliferative disorder of NK cells. Blood.

[31] Le K., Vollenweider J., Han J., Staudinger N., Stenson M., Bayraktar L., Wellik L.E., Maurer M.J., McPhail E.D., Witzig T.E. (2024). Dependence of peripheral T-cell lymphoma on constitutively activated JAK3: Implication for JAK3 inhibition as a therapeutic approach. Hematol. Oncol..

[32] Walters D.K., Mercher T., Gu T.L., O’Hare T., Tyner J.W., Loriaux M., Goss V.L., Lee K.A., Eide C.A., Wong M.J. (2006). A Activating alleles of JAK3 in acute megakaryoblastic leukemia. Cancer Cell..

[33] Kiyoi H., Yamaji S., Kojima S., Naoe T. (2007). JAK3 mutations occur in acute megakaryoblastic leukemia both in Down syndrome children and non-Down syndrome adults. Leukemia.

[34] Riera L., Lasorsa E., Bonello L., Sismondi F., Tondat F., Di Bello C., Di Celle P.F., Chiarle R., Godio L., Pich A. (2011). Description of a novel Janus kinase 3 P132A mutation in acute megakaryoblastic leukemia and demonstration of previously reported Janus kinase 3 mutations in normal subjects. Leuk. Lymphoma.

[35] Sakaguchi H., Okuno Y., Muramatsu H., Yoshida K., Shiraishi Y., Takahashi M., Kon A., Sanada M., Chiba K., Tanaka H. (2013). Exome sequencing identifies secondary mutations of SETBP1 and JAK3 in juvenile myelomonocytic leukemia. Nat. Genet..

[36] Das Gupta D., Paul C., Samel N., Bieringer M., Staudenraus D., Marini F., Raifer H., Menke L., Hansal L., Camara B. (2022). IRF4 deficiency vulnerates B-cell progeny for leukemogenesis via somatically acquired Jak3 mutations conferring IL-7 hypersensitivity. Cell Death Differ..

[37] Li S.D., Ma M., Li H., Waluszko A., Sidorenko T., Schadt E.E., Zhang D.Y., Chen R., Ye F. (2017). Cancer gene profiling in non-small cell lung cancers reveals activating mutations in JAK2 and JAK3 with therapeutic implications. Genome Med..

[38] Mittempergher L., Piskorz A.M., Bosma A.J., Michaut M., Wisman G.B.A., Kluin R.J.C., Nieuwland M., Brugman W., van der Ven K.J.W., Marass F. (2020). Kinome capture sequencing of high-grade serous ovarian carcinoma reveals novel mutations in the JAK3 gene. PLoS ONE.

[39] Greenplate A., Wang K., Tripathi R.M., Palma N., Ali S.M., Stephens P.J., Miller V.A., Shyr Y., Guo Y., Reddy N.M. (2018). Genomic profiling of T-cell neoplasms reveals frequent *JAK1* and *JAK3* mutations with clonal evasion from targeted therapies. JCO Precis. Oncol..

[40] Velatooru L.R., Hu C.H., Bijani P., Wang X., Bojaxhi P., Chen H., Duvic M., Ni X. (2023). New *JAK3-INSL3* fusion transcript-an oncogenic event in cutaneous T-cell lymphoma. Cells.

[41] Degryse S., De Bock C.E., Cox L., Demeyer S., Gielen O., Mentens N., Jacobs K., Geerdens E., Gianfelici V., Hulselmans G. (2014). JAK3 mutants transform hematopoietic cells through JAK1 activation, causing T-cell acute lymphoblastic leukemia in a mouse model. Blood.

[42] Losdyck E., Hornakova T., Springuel L., Degryse S., Gielen O., Cools J., Constantinescu S.N., Flex E., Tartaglia M., Renauld J.C. (2015). Distinct acute lymphoblastic leukemia (ALL)-associated Janus kinase 3 (JAK3) mutants exhibit different cytokine-receptor requirements and JAK inhibitor specificities. J. Biol. Chem..

[43] Vadivel C.K., Gluud M., Torres-Rusillo S., Boding L., Willerslev-Olsen A., Buus T.B., Nielsen T.K., Persson J.L., Bonefeld C.M., Geisler C. (2021). JAK3 is expressed in the nucleus of malignant T cells in cutaneous T cell lymphoma (CTCL). Cancers.

[44] Basheer F., Bulleeraz V., Ngo V.Q.T., Liongue C., Ward A.C. (2022). In vivo impact of JAK3 A573V mutation revealed using zebrafish. Cell Mol. Life Sci..

[45] Barreiros L.A., Segundo G.R.S., Grumach A.S., Roxo-Junior P., Torgerson T.R., Ochs H.D., Condino-Neto A. (2018). A novel homozygous JAK3 mutation leading to T-B+NK- SCID in two Brazilian patients. Front. Pediat..

[46] Bodaar K., Yamagata N., Barthe A., Landrigan J., Chonghaile T.N., Burns M., Stevenson K.E., Devidas M., Loh M.L., Hunger S.P. (2022). JAK3 mutations and mitochondrial apoptosis resistance in T-cell acute lymphoblastic leukemia. Leukemia.

[47] Degryse S., Bornschein S., de Bock C.E., Leroy E., Vanden Bempt M., Demeyer S., Jacobs K., Geerdens E., Gielen O., Soulier J. (2018). Mutant JAK3 signaling is increased by loss of wild-type JAK3 or by acquisition of secondary JAK3 mutations in T-ALL. Blood.

[48] Kiel M.J., Velusamy T., Rolland D., Sahasrabuddhe A.A., Chung F., Bailey N.G., Schrader A., Li B., Li J.Z., Ozel A.B. (2014). Integrated genomic sequencing reveals mutational landscape of T-cell prolymphocytic leukemia. Blood.

[49] Lahera A., Vela-Martin L., Fernandez-Navarro P., Llamas P., Lopez-Lorenzo J.L., Cornago J., Santos J., Fernandez-Piqueras J., Villa-Morales M. (2024). The JAK3(Q988P) mutation reveals oncogenic potential and resistance to ruxolitinib. Mol. Carcinog..

[50] Malinge S., Ragu C., Della-Valle V., Pisani D., Constantinescu S.N., Perez C., Villeval J.L., Reinhardt D., Landman-Parker J., Michaux L. (2008). Activating mutations in human acute megakaryoblastic leukemia. Blood.

[51] Notarangelo L.D., Mella P., Jones A., de Saint Basile G., Savoldi G., Cranston T., Vihinen M., Schumacher R.F. (2001). Mutations in severe combined immune deficiency (SCID) due to JAK3 deficiency. Hum. Mutat..

[52] Qamar F., Junejo S., Qureshi S., Seleman M., Bainter W., Massaad M., Chou J., Geha R.S. (2017). A novel mutation in the JH4 domain of JAK3 causing severe combined immunodeficiency complicated by vertebral osteomyelitis. Clin. Immunol..

[53] Roberts J.L., Lengi A., Brown S.M., Chen M., Zhou Y.J., O’Shea J.J., Buckley R.H. (2004). Janus kinase 3 (JAK3) deficiency: Clinical, immunologic, and molecular analyses of 10 patients and outcomes of stem cell transplantation. Blood.

[54] Sato T., Toki T., Kanezaki R., Xu G., Terui K., Kanegane H., Miura M., Adachi S., Migita M., Morinaga S. (2008). Functional analysis of JAK3 mutations in transient myeloproliferative disorder and acute megakaryoblastic leukaemia accompanying Down syndrome. Br. J. Haematol..

[55] Sic H., Speletas M., Cornacchione V., Seidl M., Beibel M., Linghu B., Yang F., Sevdali E., Germenis A.E., Oakeley E.J. (2017). An activating Janus kinase-3 mutation is associated with cytotoxic T lymphocyte antigen-4-dependent immune dysregulation syndrome. Front. Immunol..

[56] Stepensky P., Keller B., Shamriz O., NaserEddin A., Rumman N., Weintraub M., Warnatz K., Elpeleg O., Barak Y. (2016). Deep intronic mis-splicing mutation in JAK3 gene underlies T-B+NK- severe combined immunodeficiency phenotype. Clin. Immunol..

[57] Xiao W., Gupta G.K., Yao J., Jang Y.J., Xi L., Baik J., Sigler A., Kumar A., Moskowitz A.J., Arcila M.E. (2019). Recurrent somatic JAK3 mutations in NK-cell enteropathy. Blood.

[58] Yamashita Y., Yuan J., Suetake I., Suzuki H., Ishikawa Y., Choi Y.L., Ueno T., Soda M., Hamada T., Haruta H. (2010). Array-based genomic resequencing of human leukemia. Oncogene.

[59] Agarwal A., MacKenzie R.J., Eide C.A., Davare M.A., Watanabe-Smith K., Tognon C.E., Mongoue-Tchokote S., Park B., Braziel R.M., Tyner J.W. (2015). Functional RNAi screen targeting cytokine and growth factor receptors reveals oncorequisite role for interleukin-2 gamma receptor in JAK3-mutation-positive leukemia. Oncogene.

[60] Liu Y., Easton J., Shao Y., Maciaszek J., Wang Z., Wilkinson M.R., McCastlain K., Edmonson M., Pounds S.B., Shi L. (2017). The genomic landscape of pediatric and young adult T-lineage acute lymphoblastic leukemia. Nat. Genet..

[61] Avagyan S., Brown A.L. (2021). To T or not to B: Germline RUNX1 mutation preferences in pediatric ALL predisposition. J. Clin. Investig..

[62] de Bock C.E., Cools J. (2018). JAK3 mutations and HOXA9 expression are important cooperating events in T-cell acute lymphoblastic leukemia. Mol. Cell. Oncol..

[63] Yuan S., Wang X., Hou S., Guo T., Lan Y., Yang S., Zhao F., Gao J., Wang Y., Chu Y. (2022). PHF6 and JAK3 mutations cooperate to drive T-cell acute lymphoblastic leukemia progression. Leukemia.

[64] Broux M., Prieto C., Demeyer S., Vanden Bempt M., Alberti-Servera L., Lodewijckx I., Vandepoel R., Mentens N., Gielen O., Jacobs K. (2019). Suz12 inactivation cooperates with JAK3 mutant signaling in the development of T-cell acute lymphoblastic leukemia. Blood.

[65] Springuel L., Hornakova T., Losdyck E., Lambert F., Leroy E., Constantinescu S.N., Flex E., Tartaglia M., Knoops L., Renauld J.C. (2014). Cooperating JAK1 and JAK3 mutants increase resistance to JAK inhibitors. Blood.

[66] de Bock C.E., Demeyer S., Degryse S., Verbeke D., Sweron B., Gielen O., Vandepoel R., Vicente C., Vanden Bempt M., Dagklis A. (2018). HOXA9 cooperates with activated JAK/STAT signaling to drive leukemia development. Cancer Discov..

[67] Jamrog L., Chemin G., Fregona V., Coster L., Pasquet M., Oudinet C., Rouquie N., Prade N., Lagarde S., Cresson C. (2018). PAX5-ELN oncoprotein promotes multistep B-cell acute lymphoblastic leukemia in mice. Proc. Natl. Acad. Sci. USA.

[68] Batista C.R., Lim M., Laramee A.S., Abu-Sardanah F., Xu L.S., Hossain R., Bell G.I., Hess D.A., DeKoter R.P. (2018). Driver mutations in Janus kinases in a mouse model of B-cell leukemia induced by deletion of PU.1 and Spi-B. Blood Adv..

[69] Raivola J., Haikarainen T., Abraham B.G., Silvennoinen O. (2021). Janus kinases in leukemia. Cancers.

[70] Luo Y., Alexander M., Gadina M., O’Shea J.J., Meylan F., Schwartz D.M. (2021). JAK-STAT signaling in human disease: From genetic syndromes to clinical inhibition. J. Allergy Clin. Immunol..

[71] Gadina M., Chisolm D.A., Philips R.L., McInness I.B., Changelian P.S., O’Shea J.J. (2020). Translating JAKs to Jakinibs. J. Immunol..

[72] Quintas-Cardama A., Vaddi K., Liu P., Manshouri T., Li J., Scherle P.A., Caulder E., Wen X., Li Y., Waeltz P. (2010). Preclinical characterization of the selective JAK1/2 inhibitor INCB018424: Therapeutic implications for the treatment of myeloproliferative neoplasms. Blood.

[73] Bouchekioua A., Scourzic L., De Wever O., Zhang Y., Cervera P., Aline-Fardin A., Mercher T., Gaulard P., Nyga R., Jeziorowska D. (2014). JAK3 deregulation by activating mutations confers invasive growth advantage in extranodal nasal-type natural killer cell lymphoma. Leukemia.

[74] Degryse S., de Bock C.E., Demeyer S., Govaerts I., Bornschein S., Verbeke D., Jacobs K., Binos S., Skerrett-Byrne D.A., Murray H.C. (2018). Mutant JAK3 phosphoproteomic profiling predicts synergism between JAK3 inhibitors and MEK/BCL2 inhibitors for the treatment of T-cell acute lymphoblastic leukemia. Leukemia.

[75] Damsky W., Peterson D., Ramseier J., Al-Bawardy B., Chun H., Proctor D., Strand V., Flavell R.A., King B. (2021). The emerging role of Janus kinase inhibitors in the treatment of autoimmune and inflammatory diseases. J. Allergy Clin. Immunol..

[76] Nairismagi M., Gerritsen M.E., Li Z.M., Wijaya G.C., Chia B.K.H., Laurensia Y., Lim J.Q., Yeoh K.W., Yao X.S., Pang W.L. (2018). Oncogenic activation of JAK3-STAT signaling confers clinical sensitivity to PRN371, a novel selective and potent JAK3 inhibitor, in natural killer/T-cell lymphoma. Leukemia.

[77] Cirillo E., Giardino G., Gallo V., D’Assante R., Grasso F., Romano R., Di Lillo C., Galasso G., Pignata C. (2015). Severe combined immunodeficiency—An update. Ann. N. Y. Acad. Sci..

[78] Dvorak C.C., Haddad E., Heimall J., Dunn E., Buckley R.H., Kohn D.B., Cowan M.J., Pai S.Y., Griffith L.M., Cuvelier G.D.E. (2023). The diagnosis of severe combined immunodeficiency (SCID): The Primary Immune Deficiency Treatment Consortium (PIDTC) 2022 Definitions. J. Allergy Clin. Immunol..

[79] Tangye S.G., Al-Herz W., Bousfiha A., Cunningham-Rundles C., Franco J.L., Holland S.M., Klein C., Morio T., Oksenhendler E., Picard C. (2022). Human inborn errors of immunity: 2022 update on the classification from the International Union of Immunological Societies Expert Committee. J. Clin. Immunol..

[80] Marciano B.E., Huang C.Y., Joshi G., Rezaei N., Carvalho B.C., Allwood Z., Ikinciogullari A., Reda S.M., Gennery A., Thon V. (2014). BCG vaccination in patients with severe combined immunodeficiency: Complications, risks, and vaccination policies. J. Allergy Clin. Immunol..

[81] Wang X., Zhu L., Ying S., Liao X., Zheng J., Liu Z., Gao J., Niu M., Xu X., Zhou Z. (2023). Increased RNA editing sites revealed as potential novel biomarkers for diagnosis in primary Sjogren’s syndrome. J. Autoimmun..

[82] Candotti F., O’Shea J.J., Villa A. (1998). Severe combined immune deficiencies due to defects of the common gamma chain-JAK3 signaling pathway. Springer Semin. Immunopathol..

[83] Macchi P., Villa A., Gillani S., Sacco M.G., Frattini A., Porta F., Ugazio A.G., Johnson J.A., Candotti F., O’Shea J.J. (1995). Mutations of Jak-3 gene in patients with autosomal severe combined immune deficiency (SCID). Nature.

[84] Russell S.M., Tayebi N., Nakajima H., Riedy M.L., Roberts J.L., Aman M.J., Migone T.S., Noguchi M., Markent M.C., Buckley R.M. (1995). Mutation of JAK3 in a patient with SCID: Essential role of JAK3 in lymphoid development. Science.

[85] Candotti F., Oakes S.A., Johnston J.A., Giliani S., Schumacher R.F., Mella P., Fiorini M., Ugazio A.G., Badolato R., Notarangelo L.D. (1997). Structural and functional basis for JAK3-deficient severe combined immunodeficiency. Blood.

[86] Nosaka T., van Deursen J.M., Tripp R.A., Thierfelder W.E., Witthuhn B.A., McMickle A.P., Doherty P.C., Grosveld G.C., Ihle J.N. (1995). Defective lymphoid development in mice lacking Jak3. Science.

[87] Robinette M.L., Cella M., Telliez J.B., Ulland T.K., Barrow A.D., Capuder K., Gilfillan S., Lin L.L., Notarangelo L.D., Colonna M. (2018). Jak3 deficiency blocks innate lymphoid cell development. Mucosal Immunol..

[88] Grossman W.J., Verbsky J.W., Yang L., Berg L.J., Fields L.E., Chaplin D.D., Ratner L. (1999). Dysregulated myelopoiesis in mice lacking Jak3. Blood.

[89] Iwanami N., Mateos F., Hess I., Riffel N., Soza-Ried C., Schorpp M., Boehm T. (2011). Genetic evidence for an evolutionarily conserved role of IL-7 signaling in T cell development of zebrafish. J. Immunol..

[90] Moore F.E., Garcia E.G., Lobbardi R., Jain E., Tang Q., Moore J.C., Cortes M., Molodtsov A., Kasheta M., Luo C.C. (2016). Single-cell transcriptional analysis of normal, aberrant, and malignant hematopoiesis in zebrafish. J. Exp. Med..

[91] Castagnoli R., Delmonte O.M., Calzoni E., Notarangelo L.D. (2019). Hematopoietic stem cell transplantation in primary immunodeficiency diseases: Current status and future perspectives. Front. Pediatr..

[92] Goncalves P., Doisne J.M., Eri T., Charbit B., Bondet V., Posseme C., Llibre A., Casrouge A., Lenoir C., Neven B. (2022). Defects in mucosal immunity and nasopharyngeal dysbiosis in HSC-transplanted SCID patients with IL2RG/JAK3 deficiency. Blood.

[93] Abd Hamid I.J., Slatter M.A., McKendrick F., Pearce M.S., Gennery A.R. (2017). Long-term outcome of hematopoietic stem cell transplantation for IL2RG/JAK3 SCID: A cohort report. Blood.

[94] Kouchaki R., Abd-Nikfarjam B., Maali A.H., Abroun S., Foroughi F., Ghaffari S., Azad M. (2020). Induced pluripotent stem cell meets severe combined immunodeficiency. Cell J..

[95] Noguchi M., Yi H., Rosenblatt H.M., Filipovich A.H., Adelstein S., Modi W.S., McBride O.W., Leonard W.J. (1993). Interleukin-2 receptor γ chain mutation results in X-linked severe combined immunodeficiency in humans. Cell.

[96] Byambaa S., Uosaki H., Hara H., Nagao Y., Abe T., Shibata H., Nureki O., Ohmori T., Hanazono Y. (2020). Generation of novel Il2rg-knockout mice with clustered regularly interspaced short palindromic repeats (CRISPR) and Cas9. Exp. Anim..

[97] Sertori R., Jones R., Basheer F., Rivera L., Dawson S., Loke S., Heidary S., Dhillon A., Liongue C., Ward A.C. (2022). Generation and characterization of a zebrafish IL-2Rgc SCID model. Int. J. Mol. Sci..

[98] Puel A., Ziegler S.F., Buckley R.H., Leonard W.J. (1998). Defective IL7R expression in T-B+NK + severe combined immunodeficiency. Nat. Genet..

[99] Roifman C.M., Zhang J., Chitayat D., Sharfe N. (2000). A partial deficiency of interleukin-7R alpha is sufficient to abrogate T-cell development and cause severe combined immunodeficiency. Blood.

[100] Peschon J.J., Morrissey P.J., Grabstein K.H., Ramsdell F.J., Maraskovsky E., Gliniak B.C., Park L.S., Ziegler S.F., Williams D.E., Ware C.B. (1994). Early lymphocyte expansion is severely impaired in interleukin 7 receptor-deficient mice. J. Exp. Med..

[101] He Y.W., Malek T.R. (1996). Interleukin-7 receptor alpha is essential for the development of gamma delta + T cells, but not natural killer cells. J. Exp. Med..

[102] Hwa V. (2016). STAT5B deficiency: Impacts on human growth and immunity. Growth Horm. IGF Res..

[103] Heidary S., Awasthi N., Page N., Allnutt T., Lewis R.S., Liongue C., Ward A.C. (2023). A zebrafish model of growth hormone insensitivity syndrome with immune dysregulation 1 (GHISID1). Cell. Mol. Life Sci..

[104] Lewis R.S., Stephenson S.E.M., Ward A.C. (2006). Constitutive activation of zebrafish Stat5 expands hematopoietic cell populations in vivo. Exp. Hematol..

[105] Pham H.T.T., Maurer B., Prchal-Murphy M., Grausenburger R., Grundschober E., Javaheri T., Nivarthi H., Boersma A., Kolbe T., Elabd M. (2018). STAT5BN642H is a driver mutation for T cell neoplasia. J. Clin. Investig..

